# A 24-Hour Study of the Hypothalamo-Pituitary Axes in Huntington’s Disease

**DOI:** 10.1371/journal.pone.0138848

**Published:** 2015-10-02

**Authors:** Eirini Kalliolia, Edina Silajdžić, Rajasree Nambron, Seán J. Costelloe, Nicholas G. Martin, Nathan R. Hill, Chris Frost, Hilary C. Watt, Peter Hindmarsh, Maria Björkqvist, Thomas T. Warner

**Affiliations:** 1 Department of Clinical Neurosciences, UCL Institute of Neurology, London, United Kingdom; 2 Brain Disease Biomarker Unit, Department of Experimental Medical Science, Wallenberg Neuroscience Centre, Lund University, Lund, Sweden; 3 Biochemistry Department, Royal Free Hospital, London, United Kingdom; 4 Nuffield Department of Primary Care Health Sciences, University of Oxford, Oxford, United Kingdom; 5 Department of Medical Statistics, London School of Hygiene and Tropical Medicine, London, United Kingdom; 6 Department of Public Health and Primary Care, Imperial College, London, United Kingdom; 7 Developmental Endocrinology Research Group, UCL Institute of Child Health, London, United Kingdom; 8 Reta Lila Weston Institute of Neurological Studies, UCL Institute of Neurology, London, United Kingdom; University of Cordoba, SPAIN

## Abstract

**Background:**

Huntington’s disease is an inherited neurodegenerative disorder characterised by motor, cognitive and psychiatric disturbances. Patients exhibit other symptoms including sleep and mood disturbances, muscle atrophy and weight loss which may be linked to hypothalamic pathology and dysfunction of hypothalamo-pituitary axes.

**Methods:**

We studied neuroendocrine profiles of corticotropic, somatotropic and gonadotropic hypothalamo-pituitary axes hormones over a 24-hour period in controlled environment in 15 healthy controls, 14 premanifest and 13 stage II/III Huntington’s disease subjects. We also quantified fasting levels of vasopressin, oestradiol, testosterone, dehydroepiandrosterone sulphate, thyroid stimulating hormone, free triiodothyronine, free total thyroxine, prolactin, adrenaline and noradrenaline. Somatotropic axis hormones, growth hormone releasing hormone, insulin-like growth factor-1 and insulin-like factor binding protein-3 were quantified at 06:00 (fasting), 15:00 and 23:00. A battery of clinical tests, including neurological rating and function scales were performed.

**Results:**

24-hour concentrations of adrenocorticotropic hormone, cortisol, luteinizing hormone and follicle-stimulating hormone did not differ significantly between the Huntington’s disease group and controls. Daytime growth hormone secretion was similar in control and Huntington’s disease subjects. Stage II/III Huntington’s disease subjects had lower concentration of post-sleep growth hormone pulse and higher insulin-like growth factor-1:growth hormone ratio which did not reach significance. In Huntington’s disease subjects, baseline levels of hypothalamo-pituitary axis hormones measured did not significantly differ from those of healthy controls.

**Conclusions:**

The relatively small subject group means that the study may not detect subtle perturbations in hormone concentrations. A targeted study of the somatotropic axis in larger cohorts may be warranted. However, the lack of significant results despite many variables being tested does imply that the majority of them do not differ substantially between HD and controls.

## Introduction

Huntington´s disease (HD) is an inherited neurodegenerative disease, caused by a CAG triplet repeat expansion in the gene encoding huntingtin [1]. Classical features of HD include motor manifestations, cognitive and psychiatric symptoms [2]. However, these are not the sole manifestations in HD, and disruption of circadian rhythms [3–6], alterations in sleep patterns [7–9], altered glucose homeostasis [10–13], muscle atrophy [14] and weight loss [15–17] may also impact on the quality of life of the patients and can precede motor symptoms by many years. Extensive research in animal models of HD [18,19] and HD patients [20–26] suggests that these symptoms could be linked to progressive hypothalamic pathology and changes in the neuroendocrine systems.

The hypothalamus exerts control over many bodily functions via three major outputs: autonomic, endocrine and behavioural systems. It acts as the coordinating centre for the neuroendocrine system. Hypothalamic endocrine efferent output is mediated through the hypothalamic pituitary axes [27,28], regulating the function of the thyroid gland, the adrenal gland, and the gonads, and, thereby, the circulating levels of growth hormone, thyroid hormones, cortisol, testosterone and oestrogens [29,30].

Alterations of the hypothalamic-pituitary-adrenal (HPA) axis have been shown in HD patients [24,26,31,32] and in HD mouse models [19]. Interestingly, increased cortisol levels [22–24,26] can cause symptoms that are common in HD patients such as depression [33–36], skeletal muscle atrophy, altered glucose tolerance and memory impairment [37].

Since the thyrotropic axis is involved in the regulation of body weight and metabolism [38], which are affected in HD [15], several studies have evaluated hypothalamic-pituitary-thyroid (HPT) axis function in patients with HD, with conflicting results [24,39–41].

The hypothalamic-pituitary-gonadal (HPG) axis has also been shown altered in HD mice [42,43] and in men with HD, where reduced testosterone levels have been shown to be linked to disease severity [24,44]. Gonadotropic axis hormones have not been carefully investigated in female HD patients.

We conducted a study to analyse the corticotropic, thyrotropic, gonadotropic, somatotropic and lactotropic axes in detail over a 24-hour period in a controlled environment, using cohorts of premanifest and moderate HD subjects and healthy controls.

## Methods

### Participants

Patients were eligible for enrolment if they were 18 years of age or older, had completed either a predictive or diagnostic genetic test for HD (CAG repeat ≥40). Patients were excluded from the study if they had: pre-existent endocrine disease, central nervous system disorder other than HD, history of alcohol or drug abuse, treatment with corticosteroids, phenothiazine anti-emetics, antipsychotic medication (including neuroleptics, SSRI drugs), or hypnotic drugs for preceding 6 months, night shift working and weight change in the preceding 6 months. Controls were recruited principally from the partners, spouses, or carers of the HD group with no clinical evidence or family history of HD and the same exclusion criteria applied. Fifteen healthy controls, 14 gene carriers (premanifest HD) and 13 stage II/III HD patients were enrolled into a study analysing neuroendocrine factors. The study was conducted at the Royal Free London NHS Foundation Trust. Participants were recruited through the HD Multidisciplinary Clinic at the National Hospital for Neurology and Neurosurgery, London, UK. Written informed consent was obtained from all subjects. The study protocol was approved by the joint UCL/UCLH ethics committee and was conducted in accordance with the Declaration of Helsinki.

### Clinical protocol

Study subjects underwent to a 24-hour inpatient stay in the hospital. Subjects were advised to observe their usual bedtime, wake time, and mealtime patterns in the week preceding the study. During the study, subjects could walk freely or watch television, but not fall asleep or snack outside scheduled mealtimes. A cannula was inserted 60 minutes before the start of blood sampling at 14:00 hours. Sampling was performed through a long line to minimise sleep disruption. The aim for female subjects was to be sampled, where possible, at the midpoint of their menstrual cycle in order to standardise hormone analysis.

The following pituitary axis hormones were assayed: vasopressin, corticotropic-axis hormones (adrenocorticotropic hormone [ACTH] and cortisol), somatotropic-axis hormones (growth hormone releasing hormone [GHRH], growth hormone [GH], insulin-like growth factor-1 [IGF-1], and insulin-like factor binding protein-3 [IGF-BP3]), gonadotropic axis hormones (luteinizing hormone [LH], follicle-stimulating hormone [FSH], oestradiol and testosterone in female and male), thyrotropic axis hormones (thyroid stimulating hormone [TSH], free triiodothyronine [fT3], and free total thyroxine [fT4]) and lactotropic axis hormone (prolactin). Samples for ACTH, cortisol, GH, LH and FSH analysis were taken hourly from 14:00 on day 1 until 13:00 on day 2. Plasma for catecholamines (adrenaline, noradrenaline), vasopressin, fT3, fT4 and TSH, prolactin, total testosterone, oestradiol and dehydroepiandrosterone sulphate (DHEAS) analysis was drawn at 06:00 on day 2 after an overnight fast. Plasma for GHRH, IGF-1and IGF-BP3 was taken at 06:00, 15:00 and 23:00. During the study, urine was collected over 24 hours for the determination of cortisol concentration.

Clinical assessment and HD rating scales were performed by neurologist with expertise in HD. The Unified Huntington’s Disease Rating Scale (UHDRS), motor section (MS), Total Functional Capacity (TFC), and Functional activity (FA), were assessed. Cognitive function was assessed by Stroop Test Evaluation Colour Naming (STECN), Stroop Test Evaluation Word Reading (STEWR), Stroop Test Evaluation Interference (STEI), Symbol Digit Test (SDT) and Verbal Fluency Test (VFT). Problem behaviours assessment (PBA) was also used and behavioural score was calculated [45–47]. For HD gene carriers, subjects who were scored as 4 on the diagnostic confidence score of the Motor scale (motor abnormalities >99% likely to be due to HD) were classed as affected and those who scored 0 or 1 were classed as premanifest.

### Sample collection and analysis

Assays were performed according to the manufacturer’s instructions for the relevant kit. Blood for the analysis of serum cortisol, GH, LH, FSH, oestradiol, DHEAS, testosterone, prolactin and thyroid hormones were collected in BD Vacutainer Serum Separation Tubes while blood for ACTH analysis was collected into EDTA containing tubes. Plasma samples for catecholamines and vasopressin were collected in BD Vacutainer Lithium Heparin tubes. Specimens for ACTH analysis were immediately placed on ice and were centrifuged at 2500 rotations per minute (rpm), at 4°C, for 5 minutes, soon after venesection. Specimens for catecholamines were also spun immediately after collection while all the other specimens were kept at room temperature and centrifuged within 60 minutes of sampling at 2500 rpm, at 4°C, for 5 minutes. Plasma and serum samples were immediately frozen on dry ice and stored at ‐80°C prior to analysis.

24 hour urine samples for free cortisol measurement were collected into plain containers, the bottles were weighed and the total urine volume recorded. 600 μL aliquots of urine underwent liquid-liquid extraction with 3 mL dichloromethane. 1.5 mL of the non-polar layer was transferred into glass boiling tubes and heated at 50°C until dry. The samples were reconstituted in 300 μL Elecsys Universal Diluent (Roche) and cortisol measured using the assay described below. The 24 hour excretion values were calculated by multiplying the cortisol concentration by the 24 hour urine volume.

Serum IGF-1, IGF-BP3 and GHRH were measured in duplicate by enzyme-linked immunosorbent assays (R&D Systems Europe, Ltd. Abingdon, UK). Concentrations of cortisol (measuring range (MR): 0.5–1750 nmol/L), FSH (MR: 0.100–200 mIU/mL), LH (MR: 0.100–200 mIU/mL), FT3 (MR: 0.40–50.00 pmol/L), FT4 (MR: 0.3–100 pmol/L), TSH (MR: 0.005–100 μIU/mL), prolactin (MR: 1.00–10000 μIU/mL), total testosterone (MR: 2.50–1500 ng/dL), DHEAS (MR: 0.003–27.0 μmol/L) and oestradiol (MR: 0.003–27.0 μmol/L) were determined by electrochemiluminescent immunoassay on a Roche Modular E170 autoanalyser. Serum GH and ACTH were quantified using chemiluminescent immunoassays on a Siemens Immulite Analyser (GH MR: 0.01–40.00 μg/L, ACTH MR: 5–1250 pg/mL). Plasma catecholamines were quantified by reverse phase partition high performance liquid chromatography HPLC.

### Statistical analysis

Results are expressed as mean ± standard deviation (SD) or median (minimum-maximum). Disease burden score, which correlates with and is an indicator of the severity of neuropathology of HD, was calculated for each HD gene carrier using the formula [CAG repeat length – 35.5} x age [48]. Inter-group differences were assessed by linear regression of the relevant variable (or its log if necessary for normality) on disease group and adjusting for age and gender, or (in the absence of normality or log-normality) by using a permutation test after Freedman and Lane [49]. This latter test is based on permutations of the residuals calculated under the reduced model of the full linear regression one, with the same predictor variables [50]. For relevant sex hormones, where there are marked differences by gender, analyses were performed separately by gender. All tests were two-tailed and significance level was set at p<0.05. There was no formal adjustment for multiple testing, since all hypotheses tested are interesting in their own right, although the number of comparisons made is borne in mind in the interpretation of any significant results [51]. Statistical analyses were performed using SPSS for Windows (release 16.0, SPSS, Inc., Chicago, IL) and using Stata (release 13: StataCorp Stata Statistical Software: Release 13. College Station, TX: StataCorp LP).

Fourier Transformation (FT) analysis was utilised to measure spectral power of ACTH, cortisol, GH and LH oscillations, enabling analysis of the strength/power of hormonal oscillations at different frequencies [52,53] (http://ora.ox.ac.uk/objects/ora:2356). Signal–noise ratio was improved using a 3-point moving average and any trend in the data was removed by a difference + mean regression. Data were analysed using the EASY-TSA program (Oxford University, UK: Nathan.hill@phc.ox.ac.uk) [53]. Comparisons between waveforms of time series analysis were performed by probability of SEM at any discrete time-point overlapping the SEM of the comparative group.

## Results

The demographic data and clinical characteristics of the healthy controls, premanifest, and stage II/III HD patients are reported in Table 1. This shows some differences between groups, particularly in age, where median age is lower in the premanifest group, and older in the moderate/ severe Huntington group, compared to controls. For clinical ratings there is some overlap in range for premanifest and manifest HD groups. This reflects the fact that HD pathology is a continuum and although the diagnostic confidence score identifies the groups there is not a strict dichotomy for the two groups. For further discussion of the diagnosis and classification of HD see review by Ross *et al* [54]. Three patients with stage II/III HD were excluded on screening as they were taking neuroleptic medication. Table 2 presents hormone concentrations.

**Table 1:** Demographic characteristics and clinical features of controls, pre-manifest and stage II/III HD cohorts. Data presented as median (range).

Disease stage	Controls	Pre-manifest HD	Stage II/III HD
Number of subjects	15	14	13
Age	54 (29–69)	45 (31–58)	58 (42–70)
Female: Male	6:9	9:5	5:8
BMI	25 (20–37)	27 (23–39)	26 (20–33)
CAG	-	42 (40–47)	42 (42–47)
Disease burden score	-	299 (207–434)	410 (273–702)
Functional Assessment	25	25 (21–25)	21 (11–24)
UHDRS Total Functional Capacity	13	13 (12–13)	9 (5–12)
UHDRS Motor Score	0	0 (0–11)	38 (10–65)
STECN	82 (60–93)	71 (47–91)	45 (30–81)
STEWR	100 (83–102)	96 (64–110)	57 (37–100)
STEI	45 (29–68)	42 (34–52)	24 (20–44)
SDT	51 (42–65)	52 (37–62)	31 (17–50)
VFT	48 (30–66)	41 (26–70)	27 (12–58)
CS	322 (276–359)	298 (225–369)	182 (133–308)
PBA	2 (0–10)	8 (2–31)	21 (5–55)

**Table 2:** Hormone levels in control, premanifest and stage II/III HD cohorts. Data are presented as Mean ±SD for normally distributed data and as median [minimum–maximum] for skewed data.

Axis	Control	Premanifest HD	Stage II/III HD	*p*
**Corticotropic axis**				
ACTH, 24 h, ng/L	11.4 [7.4–23.5]	13.0 [7.7–28.0]	13.4 [7.1–31.4]	0.63^L^
Cortisol, 24 h, nmol/L	232 [165–636]	224 [100–498]	212 [97–363]	0.54^L^
Cortisol, urine, nmol/24 h	179 [58–758]	136 [35–434]	121 [50–295]	0.63^L^
Cortisol: ACTH ratio, 24 h	79200 [7800–802000]	63300 [9100–489300]	65100 [10600–317200]	0.35^L^
Cortisol: ACTH ratio, evening	93600 [37000–705200]	70800 [25300–226900]	60800 [22900–142500]	0.06^L^
**Vasopressin**, pg/ml	0.84 [0.56–1.92]	0.79 [0.59–1.49]	0.77 [0.53–1.68]	0.89^LP^
**Somatotropic axis**				
GH, μg/L				
24 h	0.7 [0.1–5.7]	0.7 [0.1–1.6]	0.4 [0.1–1.7]	0.48^LP^
Post-sleep	2.1 [0.2–8.4]	1.1 [0.2–3.5]	0.3 [0.1–2.2]	0.09^L^
GHRF, ng/ml				
06:00	0.77 ± 0.24	0.76 ± 0.16	0.71 ± 0.23	0.7
15:00	0.76 ± 0.18	0.80 ± 0.38	0.82 ± 0.19	0.83^P^
23:00	0.78 ± 0.17	0.79 ± 0.16	0.72 ± 0.18	0.29
IGF-1, ng/ml				
06:00	106 [76–154]	121 [69–218]	90 [66–232]	0.88^L^
15:00	128 [77–154]	110 [65–230]	109 [69–250]	0.70^L^
23:00	107 [75–161]	113 [64–257]	106 [76–207]	0.997^L^
IGF-BP3, ng/ml				
06:00	2590 ± 400	2481 ± 584	2308 ± 531	0.38^P^
15:00	2823 ± 575	2444 ± 407	2625 ± 553	0.08^P^
23:00	2501 ± 557	2499 ± 358	2254 ± 494	0.50^P^
IGF-1:GH ratio				
06:00	801 [77–2194]	352 [11–2211]	987 [49–1642]	0.80^LP^
15:00	744 [31–3070]	613 [20–2451]	1389 [17–3043]	0.96^LP^
23:00	271 [15–1505]	300 [20–5132]	1149 [26–2630]	0.23^L^
**Gonadotropic axis**				
LH, U/L				
Female, 24 h	14.0 [1.6–46.5]	7.2 [0.1–30.9]	21.8 [3.2–32.1]	0.87^L^
Male, 24 h	5.2 [2.5–7.8]	5.0 [3.1–6.1]	4.9 [3.4–6.0]	0.98^L^
FSH, U/L				
Female, 24 h	46.1 [1.5–140.1]	5.0 [0.6–278.9]	32.9 [7.3–65.3]	0.88^L^
Male, 24 h	5.9 [2.4–12.0]	5.5 [3.1–6.0]	5.3 [1.8–7.4]	0.53^L^
Testosterone, nmol/L				
Females	0.73 ± 0.27	0.84 ± 0.38	0.50 ± 0.27	0.6
Males	15.5 ± 7.7	15.5 ± 5.3	16.8 ± 4.6	0.59
Oestradiol, pmol/L				
Females	50 [50–1432]	171 [50–791]	84 [56–218]	0.89^LP^
Males	109 [50–156]	100 [71–334]	121 [50–221]	0.57^L^
DHEAS, umol/L	4.81 ± 2.21	4.12 ± 1.92	2.77 ± 2.31	0.04
Females	3.7 ± 1.2	3.3 ± 1.3	2.1 ± 1.4	0.24
Males	5.6 ± 2.4	6.0 ± 1.8	3.2 ± 2.7	0.17
**Lactotropic axis**				
Prolactin, mU/L				
Females	360 ± 125	432 ± 195	461 ± 257	0.33^P^
Males	245 ± 98	247 ± 47	235 ± 60	0.98
**Thyrotropic axis**				
TSH, mU/L	2.61 ± 1.34	3.23 ± 3.01	1.98 ± 1.10	0.81
fT3, pmol/L	4.48 ± 0.70	5.04 ± 0.64	4.91 ± 0.59	0.08
fT4, pmol/L	14.7 ± 2.19	15.4 ± 2.31	15.5 ± 2.2	0.62
fT4:fT3 ratio	3.32 ± 0.42	3.09 ± 0.41	3.21 ± 0.54	0.52
**Catecholamines**				
Adrenaline, nmol/L	0.46 ± 0.17	0.36 ± 0.17	0.42 ± 0.21	0.29
Noradrenaline, nmol/L	1.86 ± 0.71	2.16 ± 0.63	1.89 ± 0.80	0.64

P-value = p-value from linear regression of specified variable on age and gender (or else by gender as indicated), L indicates that logs were taken of the specified variable prior to linear regression; P indicates Freedman and Lane permutation tests were performed to account to non-normality, LP indicates Freedman and Lane permutation tests were performed after taking logs of the specified variable. In all cases, age and gender were adjusted for.

### Corticotropic axis

**Fig 1:**
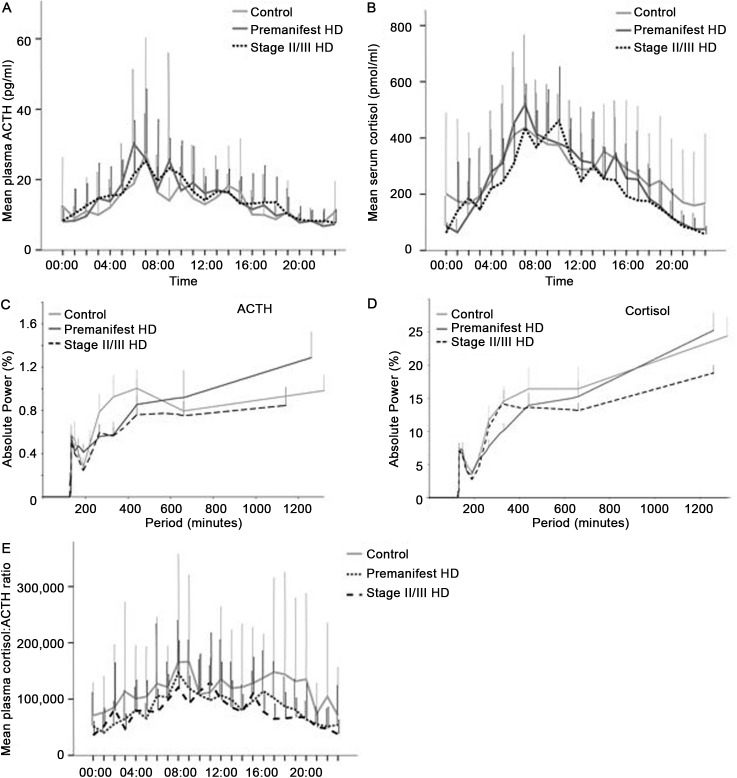
Analysis of ACTH and cortisol in control, premanifest and stage II/III HD cohorts. A: Mean ACTH concentrations over 24 hour sampling period in the three groups. B: Mean ACTH concentrations over 24 hour sampling period in the three groups. C: FT analysis of ACTH plotting strength/power (%) against frequency (minutes) of ACTH oscillations for the three groups. D: FT analysis of cortisol plotting strength/power (%) against frequency (minutes) of cortisol oscillations for the three groups. E: Mean molar cortisol:ACTH ratio over 24 hour sampling period in the three groups.

In all three groups the 24-hour profiles of ACTH and cortisol display a typical pattern with early morning peak concentrations and declining levels throughout daytime, with lowest values around midnight (Fig 1A and 1B). There was an elevation of ACTH (Fig 1A) and cortisol (Fig 1B) levels during late sleep in all three groups.

Relative FT analysis of ACTH levels showed an initial pulsatility at 132 minutes for all groups (Fig 1C) (oscillatory power: control 0.57, SEM 0.10; premanifest HD 0.50, SEM 0.10; stage II/III HD 0.57, SEM 0.09) with a further periodicity peak at 440 minutes (oscillatory power: control 1.0, SEM 0.17; premanifest HD 0.76, SEM 0.17; stage II/III HD 0.85, SEM 0.23). Overall between-group comparison at both 132 and 440 minutes did not reach statistical significance. For cortisol (Fig 1D), initial dominant pulsatility was at 132 minutes (oscillatory power: control 7.5, SEM 0.88; premanifest HD 6.31, SEM 1.16; stage II/III HD 7.04, SEM 1.17) with a further periodicity peak at 440 minutes (oscillatory power: control 16.42, SEM 3.22; premanifest HD 13.94, SEM 2.02; stage II/III HD 13.64, SEM 1.98) and again there was no significant difference between groups.

Mean levels of ACTH and cortisol over 24 hours (Table 2) did not differ significantly between groups, nor did 24-hour urinary cortisol. A comparison of cortisol to ACTH ratio for the three groups (Fig 1E) also did not demonstrate any significant differences.

### Vasopressin

We measured baseline plasma levels of vasopressin. There was no significant difference in plasma vasopressin levels between the groups.

### Somatotropic axis

**Fig 2:**
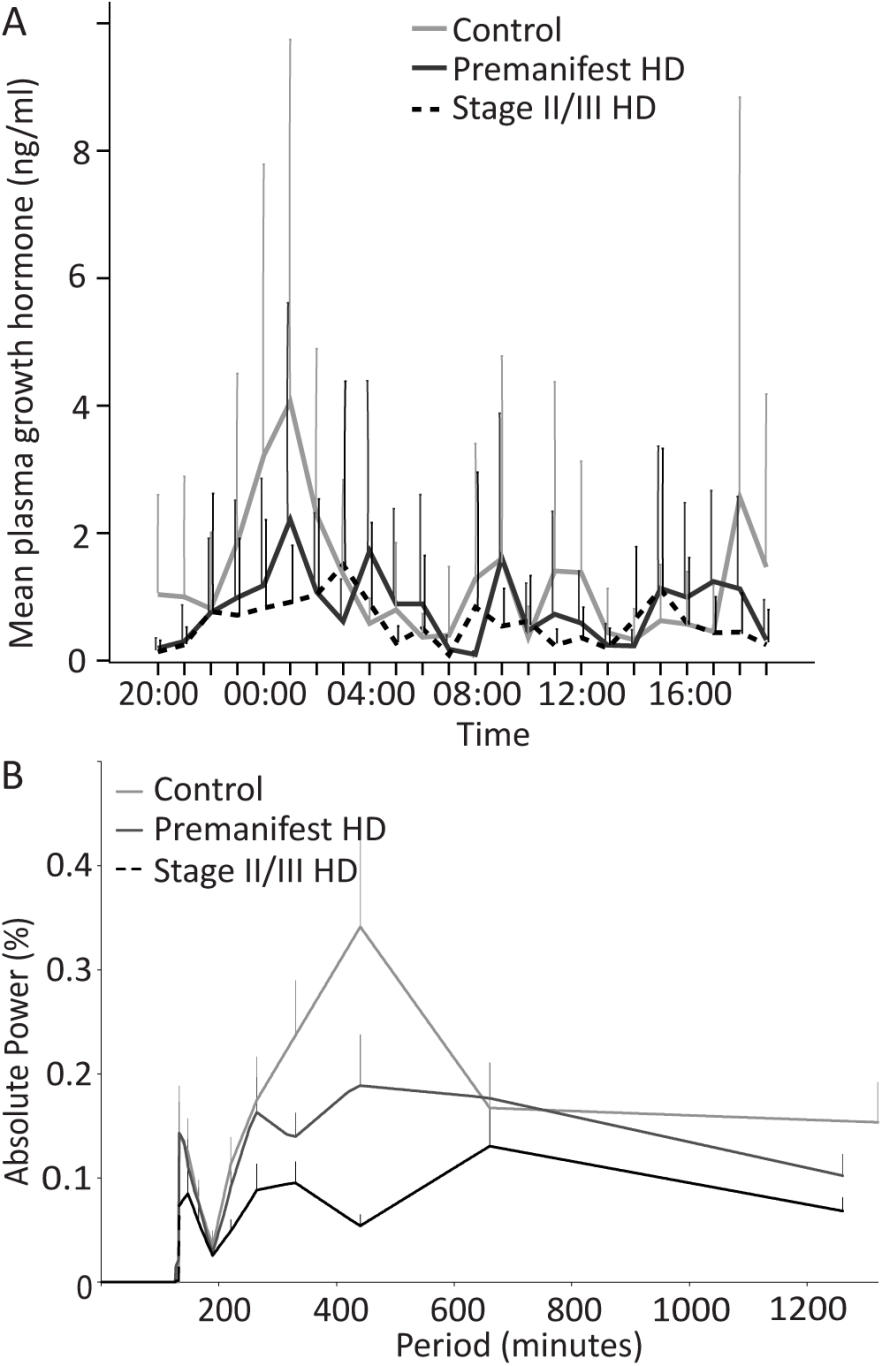
Analysis of GH in control, premanifest and stage II/III HD cohorts. A: Mean GH concentrations over 24 hour sampling period in the three groups. B: FT analysis of GH plotting strength/power (%) against frequency (minutes) of GH oscillations for the three groups.

24-hour profile of GH, and baseline levels of GHRH (a hormone that stimulates GH secretion from the pituitary), IGF-1 and IGF-BP3 are shown in Fig 2A and Table 2. In all three groups, the 24-hour profile of GH plasma levels consisted of low levels interrupted by bursts of secretion (Fig 2A). It has been reported that GH is released in a pulsatile manner with the largest and most reproducible pulse occurring shortly after sleep onset [55]. Since the subjects in this study retired to bed at 22:00, we calculated the mean GH concentration during the first half of the night (22:00–01:00). Mean concentrations of 24 hour and post-sleep GH were lower in the stage II/III HD patients but analysis corrected for age and gender did not demonstrate a significant difference (Table 2).

Relative FT of GH levels showed an initial pulsatility at 132 minutes for all groups (Fig 2B) (oscillatory power: control 0.14, SEM 0.04; premanifest HD 0.07, SEM 0.01; stage II/III HD 0.14, SEM 0.03) with a further periodicity peak at 440 minutes (oscillatory power: control 0.34, SEM 0.11; premanifest HD 0.05, SEM 0.01; stage II/III HD 0.18, SEM 0.05). Overall between-group comparison at 132 minutes did not reach statistical significance.

We also evaluated other variables of the somatotropic axis hormones, including GHRH, IGF-1 and IGF-BP3 at 06:00 (fasting), 15:00 and 23:00. There was no significant difference in plasma GHRH, IGF-1, IGF-BP3 or the IGF-1:GH ratio among the three groups at the three time-points we sampled (Table 2).

### Gonadotropic axis

We assessed the 24-hour profiles of gonadotropic axis hormones LH and FSH, as well as fasting levels of testosterone, oestradiol and DHEAS in serum from control, premanifest and stage II/III HD subjects. Since gender has a major effect on the levels of gonadotropic axis hormones, the data for all gonadotropic hormones was analysed separately for males and females.

**Fig 3:**
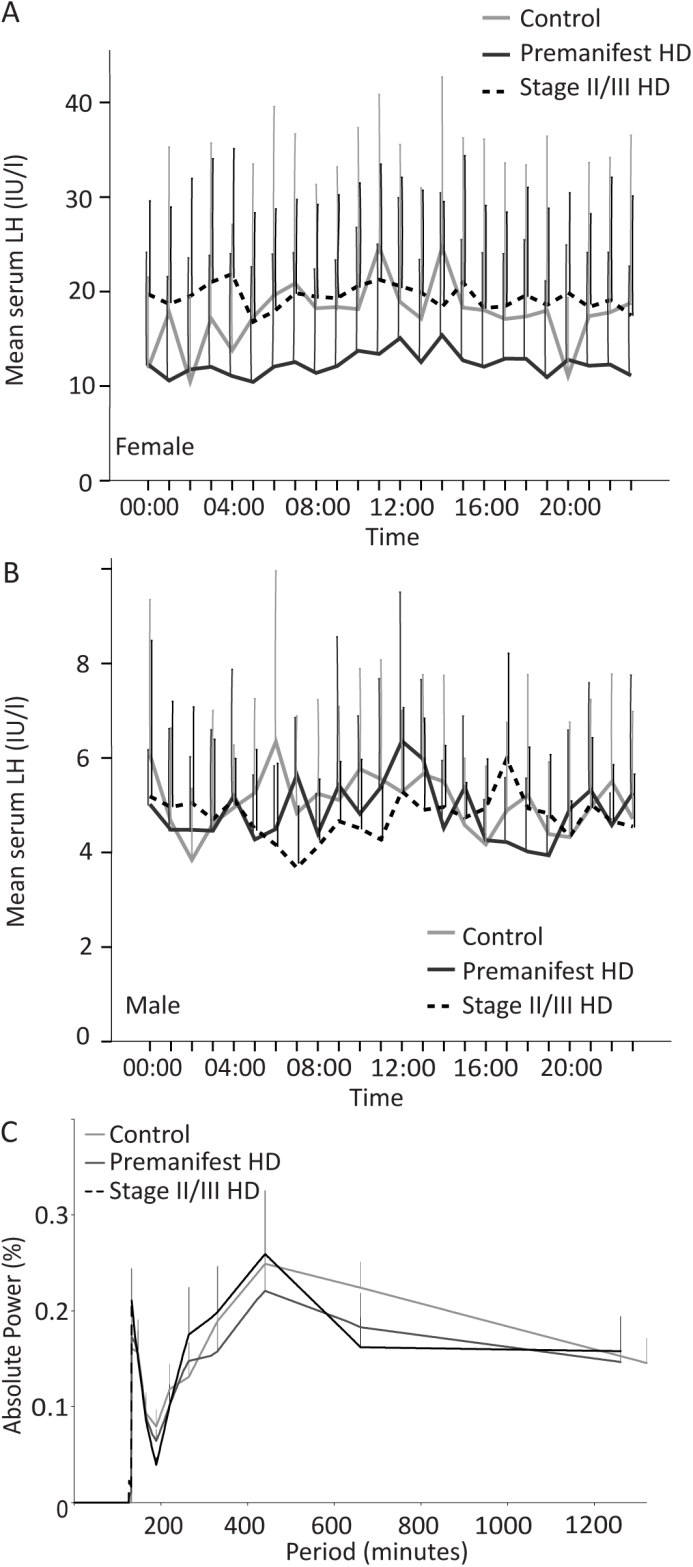
Analysis of LH in control, premanifest and stage II/III HD cohorts. A: Mean LH concentrations over 24 hour sampling period for female subjects in the three groups. B: Mean LH concentrations over 24 hour sampling period for male subjects in the three groups. C: FT analysis of LH plotting strength/power (%) against frequency (minutes) of LH oscillations for the three groups.

**Fig 4:**
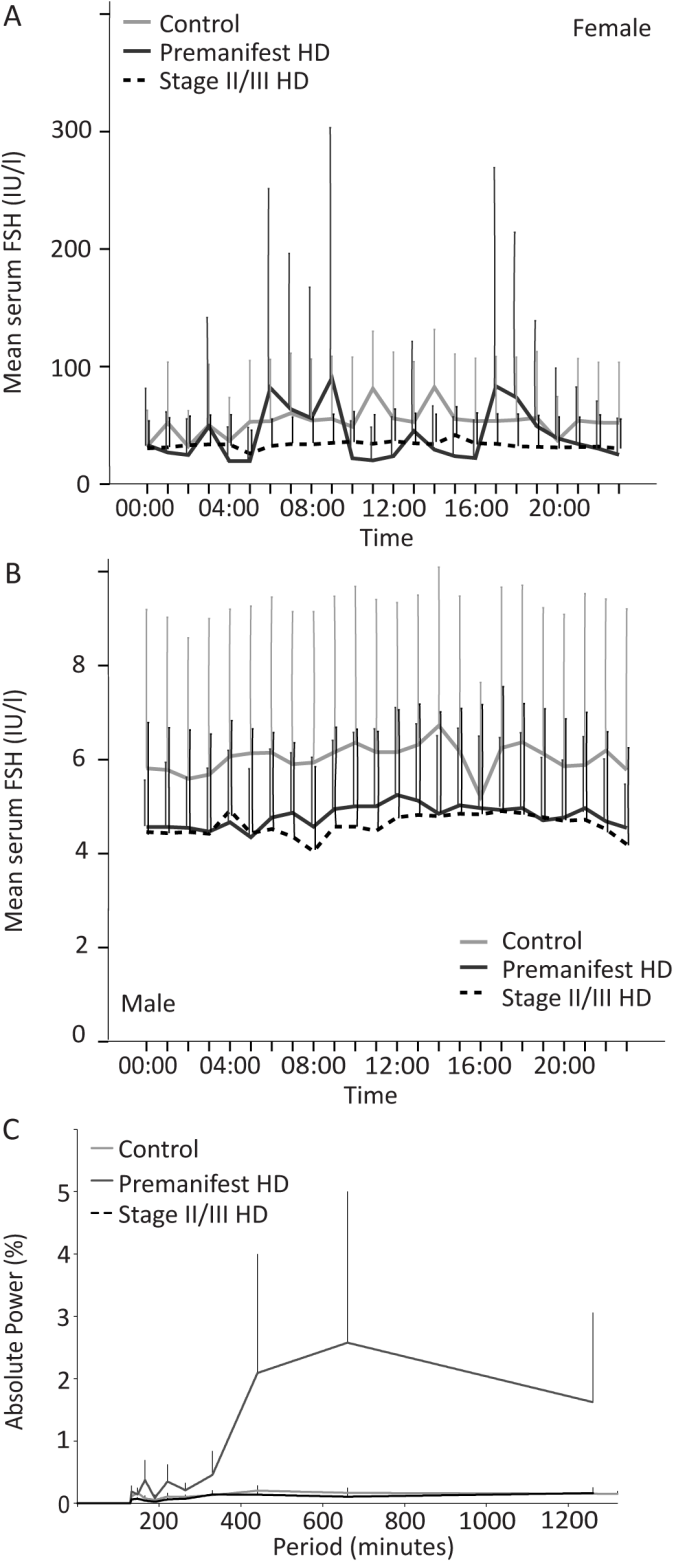
Analysis of FSH in control, premanifest and stage II/III HD cohorts. A: Mean FSH concentrations over 24 hour sampling period for female subjects in the three groups. B: Mean FSH concentrations over 24 hour sampling period for male subjects in the three groups. C: FT analysis of FSH plotting strength/power (%) against frequency (minutes) of FSH oscillations for the three groups.

In males, the pattern of LH release in all three groups exhibited episodic pulses with large inter-individual variability and no obvious diurnal variation (Fig 3B). The 24-hour FSH profile in males was similar in all three groups with very low levels throughout the day, without any diurnal variation (Fig 4B).

Although the aim for female subjects was to be sampled, where possible, at the midpoint of their menstrual cycle in order to standardise hormone analysis, this was not always the case. The young female participants were using different contraceptive methods (pill, implant or injections). Other female participants were either at a premenopausal stage with irregular menstrual cycle or menopausal. Therefore, the 24-hour LH and FSH profiles for female subjects may not be very informative. However, it can be observed that the LH and the FSH 24-hour profiles do not differ between control, premanifest HD and stage II/III HD females (Figs 3A and 4A, respectively).

Relative FT analysis of LH release showed an initial pulsatility at 132 minutes (Fig 3C) (oscillatory power: control 0.16, SEM 0.02; premanifest HD 0.21, SEM 0.03; stage II/III HD 0.17, SEM 0.05) with a further periodicity peak at 440 minutes (oscillatory power: control 0.25, SEM 0.04; premanifest HD 0.26, SEM 0.07; stage II/III HD 0.22, SEM 0.05). Overall between-group comparison at 132 and 440 minutes did not reach statistical significance.

Relative FT analysis of FSH release showed oscillatory power was supressed in both the control and stage II/III HD group in comparison to the premanifest HD group although this did not reach statistical significance. There was an initial pulsatility at 147 minutes for all groups (Fig 4C) (oscillatory power: control 0.17, SEM 0.09; premanifest HD 0.14, SEM 0.07; stage II/III HD 0.07 SEM 0.03) with a further periodicity peak in the premanifest HD group only at 660 minutes (oscillatory power: 2.58, SEM 2.43). Overall between-group comparison at 147 and 660 minutes periodicity did not reach statistical significance.

There was no significant difference in oestradiol or testosterone levels between the three groups in males or females (Table 2). Linear regression of disease group adjusting for age and sex suggested a significant difference in fasting DHEAS levels (males and females combined, F_2,36_ = 3.51, p 0.04); the means observed in the three groups shows that means were substantially lower in stage II/III HD, than in controls (with mean in premanifest HD nearer to those in controls). Analysis of DHEAS concentrations in male and female subjects separately showed lower levels in stage II/III disease groups but no significant difference in comparison with controls/premanifest HD groups (Table 2).

### Lactotropic axis

Fasting serum levels of prolactin were not significantly different between the three study cohorts (Table 2).

### Thyrotropic axis

Fasting concentrations of thyrotropic axis hormones TSH, fT3 and fT4 and ratio of fT4:fT3 in control, premanifest HD and stage II/III HD serum showed that TSH and fT4 were similar across the three groups (Table 2), and linear regression analysis adjusting for age and gender showed was no significant between group difference.

### Catecholamines

Analysis of fasting plasma concentrations of catecholamines (adrenaline and noradrenaline) did not demonstrated significant differences between the groups (Table 2).

## Discussion

Huntington’s disease (HD) is a devastating neurodegenerative disorder for which the underlying pathophysiologic mechanisms affecting the brain and other organs remain unclear. Analyses of peripheral tissues from HD patients suggest that the molecular mechanisms by which mutated huntingtin leads to cell dysfunction may be similar with those in the central nervous system [56].

The hypothalamus is an important site for pathology in HD. MRI studies have reported hypothalamic atrophy in early stage HD patients as well as in transgenic HD mice [57–61]. Grey matter alterations in the hypothalamus are among the earliest features detected in HD [62]. Microglial activation and dopamine D2 receptor reduction occur in the hypothalamic region before onset of motor symptoms in HD gene carriers [63]. Furthermore, reduction in orexin and loss of orexigenic neurons in the hypothalamus [64] and alterations in the HPA axis have been shown in mouse models and patients with HD from an early disease stage [15,19,24,26,64]. In bacterial artificial chromosome-mediated transgenic HD mice, a metabolic phenotype including impaired glucose metabolism, insulin and leptin resistance was identified that could be reversed by inactivation of mutant huntingtin in the hypothalamus [65].

A number of studies describe the effect of HD on the function of the hypothalamic-pituitary axes. However, many of these report conflicting data and are difficult to compare due to methodological issues such as different sampling times, analysis of patients with different disease stages, inclusion of patients on medication that can affect hormone levels and fasting status. In addition, with the exception of two studies that quantified vasopressin [66] and prolactin [67], previous studies did not include premanifest HD gene carriers, and thus do not inform on the state of the hypothalamic-pituitary axis early on in the disease course.

The strength of our study is that we included both premanifest and manifest HD gene carriers, and have measured all the hormones in the same cohorts during a standardised day allowing a direct comparison of hormone levels. Most previous studies applied a single or a few baseline measurements, which is inadequate to assess either the pulsatile or circadian nature of hormonal secretion, which are essential for normal hormone function [68]. We therefore performed a detailed study of hormone secretory dynamics for the main hypothalamo-pituitary axes in premanifest HD gene carriers, moderate HD patients and controls, to identify potential HD state markers and inform on neuroendocrine abnormalities associated with HD. Due to the intensive nature of this study on participants, cohort sizes were relatively small which meant that small but biologically relevant changes could be overlooked.

### Endocrine dysfunction in HD

There is evidence of abnormal function of the hypothalamus [64], pituitary gland [18], pancreatic islets [69,70] and adrenal glands [71] in animal models of HD, and to some extent in HD patients. This may lead to dysfunction of the neuroendocrine system with downstream effects on the metabolism of various cell types, including muscle cells and adipocytes, contributing to peripheral tissue abnormalities observed in HD such as weight loss, energy metabolism changes and altered glucose homeostasis [15–17].

### Corticotropic axis

In the R6/2 mouse model increased adrenal cortex volume is seen from the age of 7 weeks [71] and adrenal glands in 12-week-old mice contained intranuclear huntingtin-inclusions and weighed 37% more than in WT mice [71]. This adrenal cortical hypertrophy may explain the increased serum and urine corticosterone detected in R6/2 mice [19].

Corticotropic axis hormones ACTH and cortisol are released in pulsatile fashion with circadian and ultradian rhythms governing their secretion [72–76]. Both are released approximately hourly, with ACTH pulses preceding cortisol by approximately 10 minutes [74–76].

In HD, ACTH levels have been reported to be both increased [22] and unchanged [24]. Saleh *et al* found no significant difference between HD patients and controls in fasting morning ACTH levels [24]. Heuser *et al* found significantly higher basal cortisol and ACTH concentrations in HD patients than controls (at 19:00 hours), HD subjects had a tendency towards blunted ACTH in response to CRH stimulation, but released normal amounts of cortisol, suggesting dysregulation of the HPA axis [22].

We measured 24-hour secretion of ACTH and cortisol at hourly intervals and found no significant difference between HD patients and controls in ACTH levels over 24 hours. In agreement with Saleh *et al*, fasting levels of ACTH in the morning were not significantly different between HD patients and controls. In contrast to Heuser *et al*, we did not see increased ACTH levels in the evening, although we did not include late stage HD patients, which could explain why we do not see a significant increase in ACTH levels in the evening.

Previous studies have consistently reported that morning levels of cortisol are increased in HD patients [23,24,26], whereas one study reported similar levels in the evening [21]. While we found no significant difference in cortisol levels in HD subjects compared to controls over 24 hours, we observed higher cortisol levels in HD gene carrier in the morning but this was not statistically significant. It is possible that we would need a higher number of subjects to replicate the previously reported increase in morning cortisol.

Aziz *et al* measured cortisol levels during a 24-hour period at 10 minutes intervals [26] and found that the total cortisol secretion rate and the amplitude of the diurnal cortisol profile were significantly higher in early HD patients compared with controls. Transient awakenings interrupting sleep consistently trigger pulses of cortisol secretion [77–79] and nocturnal arousals increase morning cortisol levels [80]. It is therefore possible that the frequent sampling regime contributed to the increased cortisol levels observed.

Studies have also shown that approximately 30% of patients with depression have increased cortisol secretion [33,35,36,76] and this may be a factor contributing to increased levels seen in other HD studies. We are not able to comment from our data as no participant had significant depression.

### Vasopressin

Vasopressin is released in a circadian pattern by neurons of the hypothalamus. It plays a key role in homeostasis by the regulation of water retention through diuresis and thirst. Other roles of vasopressin include regulation of temperature, blood pressure, social behaviour and sexual motivation. Neuropathological analysis of the hypothalamus in post-mortem brain tissue has revealed a reduction in the number of vasopressin-expressing neurons [81], and interestingly was also present in a case of early stage HD who died of unrelated causes [82]. A previous study of post-mortem hypothalamic tissue showed no significant difference in the number of vasopressin neurons or vasopressin expression between controls and HD patients [83]. However, in the R6/2 mouse model the number of immunoreactive vasopressin neurons in the paraventricular nucleus of the hypothalamus were significantly decreased, suggesting that the change in drinking behaviour seen in mice may be the result of hypothalamic dysfunction [66]. In the same study patients with HD had increased thirst, unaffected urine osmolality and increased serum vasopressin, suggesting a dysregulation in the control of hypothalamic vasopressin release [66]. In another transgenic mouse model the hypothalamic expression of vasopressin was down-regulated without observing a significant loss of hypothalamic neurons [84]. In our study there was no significant difference in the fasting levels of plasma vasopressin levels between premanifest HD, stage II/III HD patients and controls. There are several differences between our study and that of Wood *et al* [66] that could account for this difference: 1. vasopressin levels differ in serum and plasma; 2. Wood *et al* included patients taking medication that may influence hormone levels; 3. Since vasopressin has a circadian rhythm [85], levels observed will depend on time of sampling: our sampling time was at 06:00 hours, whereas the sampling time in the Wood *et al* study was in the afternoon between 14:00–17:00 [66]; 4. Since hypoglycaemia causes the release of vasopressin [86], the fasting status may also have an effect on the levels of vasopressin.

### Somatotropic axis

Studies analysing GH concentration in HD have yielded conflicting results with four studies reporting no significant difference between HD patients and controls [87–90], and three studies reporting an increase in HD [24,25,91]. GH exerts its effects by stimulating IGF-1 release from the liver. Saleh *et al* [24] have reported an increase in both central (GH) and peripheral (IGF-1) somatotropic hormones in early HD patients compared to healthy controls, as well as increase with disease severity. The study included a large cohort of patients (n = 217), but only a single morning sample in the morning was analysed and the cohorts were not controlled for medication (56% patients used neuroleptics, 61% antidepressants, 37% tranquillizers) [24]. This is important since neuroleptic treatment can influence GH levels by altering dopaminergic regulation [92]. Our study excluded patients using neuroleptics in the preceding 6 months.

Phillipson *et al* [25] also reported elevated fasting plasma GH levels in HD patients compared to controls. In our study, we found no significant difference in morning, fasting levels of GH or IGF-1, which is in agreement to several previous studies [87–90]. In a 24-hour study of plasma GH concentration in female HD patients free from centrally-active medication and matched controls, Durso *et al* showed increased levels of GH in HD females throughout the 24-hour period [91], which is in contrast to our findings. However, it is known in women that the amplitude of GH secretory pulses correlate with circulating levels of oestradiol [93,94]. In our study many of the female subjects had low oestradiol levels, which may lead to the low GH concentrations. In addition, in menstruating women, 24-hour GH levels are higher compared to age-matched men [93]. Thus, since in our study we had both males and females, this may account for the lower levels of GH observed compared to the Durso *et al* study [91]. However, when we analyse the female data separately, we still do not see an increase in GH levels in HD patients.

In this study we observed lower concentrations of 24 hour and post-sleep GH in stage II/III HD patients but these were not significant. Potentially, a study of larger cohorts may verify whether this is a biologically significant observation. Decreased concentrations of post-sleep GH in premanifest and stage II/III HD subjects may be indicate delay in sleep onset since GH secretion in early sleep is temporally and quantitatively associated with the amount of slow wave sleep [95,96]. Transient awakenings during sleep inhibit GH release [97], suggesting the decrease in nocturnal GH levels in HD subjects are secondary to fragmented sleep. GHRH inhibits sleep as well as GH secretion [98], thus if decreased GH levels in the HD subjects are associated with sleep delay, one would expect GHRH release to also be lower. We did not observe any change in GHRH levels, but this did not include measurement of post-sleep levels. The normality of GHRH levels at 06:00, 15:00 and 23:00 is in keeping with the finding of no significant difference on GH concentration at these time points.

IGF-1 decreases GH release from the pituitary by negative feedback, implying that low post-sleep GH levels in HD subjects would be associated with higher IGF-1 levels. The relationship between IGF-1 and GH may be better reflected by the ratio of the two in serum at any particular time and analysis of our data showed a higher IGF-1:GH ratio in stage II/III HD patients at 23.00 but this was not statistically significant. Further studies examining sleep quality in HD patients and measuring levels of GHRH and IGF-1, in parallel with GH in larger groups may be helpful. Another reason for possible reduction in sleep-related GH levels in HD gene carriers may be linked to a reduction in the night-time melatonin release we observed in these cohorts [99], since it has been shown that melatonin administration increases basal GH release and GH responsiveness to GHRH, possibly by inhibiting endogenous somatostatin release at the hypothalamic level [100,101].

### Gonadotropic axis

In men with HD, reduced testosterone concentrations linked to disease severity have been reported [24,44]. In R6/2 mice, reduced testosterone is found and is accompanied by a reduction of gonadotropin releasing hormone (GnRH) neurons in the hypothalamus, suggesting this may be secondary to hypothalamic dysfunction [42]. In contrast, YAC128 mice develop testicular degeneration before levels of testosterone decrease and before loss of GnRH neurons in the hypothalamus can be detected, suggesting that testicular pathology results from a direct toxic effect of mutant huntingtin in the testes [43]. In contrast to previous studies, we did not observe an alteration in testosterone levels in HD males, nor in the levels of LH, FSH or oestradiol. The studies that reported lower testosterone levels in HD [24,44] included patients at different disease stages (TFC 0–13, and motor score 1–110), whereas, in our study, we had a mixture of premanifest and stage II/III HD subjects (TFC 5–13 and motor score 0–65).

To date, only two studies have analysed LH and FSH levels in male HD patients [24,44]. Both studies found no significant difference in FSH levels between control and HD males [24,44], which is in agreement with our data. Saleh *et al* found no significant difference in fasting, morning levels of LH [24], whereas Markianos *et al* reported lower LH levels in HD males in samples obtained between 10:00 and 12:00 [44]. We found no significant difference in LH levels between HD and control subjects (males or females) over 24 hours.

Gonadotropic axis hormones have not been carefully investigated in female HD patients. In our study, information on menopausal and menstrual cycle phase at the time of blood sampling for was available. We observed increased LH and FSH levels in pre- and post-menopausal women compared to other females, however, there was no significant difference between HD females and control females. More detailed study of gonadotrophic hormones at different phases of the menstrual cycle are needed to properly evaluate whether these hormones are altered in females with HD.

In the current study the only significant difference found in the gonadotropic axis was in fasting DHEAS levels, where a significant reduction could be seen in stage II/III patients with HD. This is in agreement with two previous studies that reported reduced DHEAS levels in HD patients [21,23]. Here, DHEAS was measured at only one timepoint, thus, measuring the circadian rhythm of DHEAS may be of interest. We also observed a non-significant reduction in DHEAS levels with increasing disease burden. DHEAS should ideally be analysed separately in males and females [102], however, we do not have a high number of subjects and when analysed separately for males and females, DHEAS levels are lower in HD gene carriers, however this is not statistically significant.

### Lactotropic axis

Politis *et al* 2008 demonstrated D2 receptor loss in the hypothalamus of both early-stage HD patients and premanifest HD mutation carriers [63]. D2 receptors stimulation inhibits prolactin release [103]. Prolactin levels in patients with HD have been reported to be unchanged [24,39,41,87–89,91], increased [67,104] or even decreased [105,106]. In a 24-hour study with 6 medication-free male patients, Aziz *et al* (2010) demonstrated that prolactin secretion tended to be non-significantly higher in HD patients and significantly more irregular[39]. In our study we did not detect any significant difference in fasting prolactin levels between control and HD individuals. To date it remains unclear whether the lactotropic axis is indeed affected in HD.

Prolactin rhythmicity is primarily regulated by the sleep-wake cycle and maximal secretion occurs when sleep and circadian effects are superimposed [107–109]. Like GH, increased prolactin secretion is temporally associated with slow wave sleep and fragmented sleep results in decreased prolactin levels [110].

### Thyrotropic axis

Progressive weight loss and muscle wasting are common in HD patients [14–16,111–114]. Since the thyrotropic axis is involved in the regulation of body weight and metabolism [38], several studies have evaluated hypothalamic-pituitary-thyroid axis function in patients with HD. Many studies have shown similar levels of TSH, total T4, T3 and free T4 in HD patients and normal controls [24,39–41,88]. However, in a retrospective study of HD patients, levothyroxine was found to be the most commonly prescribed drug for problems ‘unrelated’ to HD [115].

Although TSH synthesis and secretion are primarily controlled by the stimulatory action of thyrotropin-releasing hormone (TRH) and the negative feedback by thyroid hormones, other factors such as dopamine exert important modulatory effects [38]. Dopamine inhibits TSH synthesis and release through D2 receptor activation, whereas it stimulates TRH secretion [38].

One study showed similar TSH and T4 levels but higher T3 levels in early HD subjects which were not significantly increased compared with healthy controls [39]. Our findings do not support mild thyrotropic axis hyperactivity in HD but indicate a more focussed analysis in a larger cohort is warranted.

Since we only measured TSH at one time-point in the morning, we do not know whether its circadian rhythmicity is affected in HD subjects since TSH levels are low and relatively stable throughout the daytime and begin to increase in the early evening, with maximal levels around the beginning of the sleep period [116]. TSH levels progressively decline during the latter part of sleep, and this decrease is associated with slow wave sleep, while awakening result in increased TSH levels [117–119]. It has been suggested that the TSH evening rise shifts in concordance with the melatonin rhythm [120] and since we observed a reduction in the night-time melatonin in our HD cohorts [99], it is possible that TSH rhythm is also affected. Also, since the fT3 diurnal rhythm parallels TSH variations [121], the fT3 rhythmicity may also be altered in HD.

### Correlation with clinical measures

It has previously been shown in a longitudinal study that IGF-1 correlates with cognitive measures: Stroop Word Reading, Stroop Colour Naming, Symbol Digit Modalities Test and Verbal Fluency [122]. We did not observe significant correlations between IGF-1 and any of these cognitive measures. Although the correlations in our study were not significant, they were all negative. This may reflect a true biological finding although may also reflect low number of HD subjects in our study. Thus, a longitudinal follow-up of our patients is needed to elucidate whether high IGF-1 levels correlate with poor cognition.

## Conclusion

In contrast to many previous studies we describe overwhelmingly negative results, with no statistically significant differences. This may in part be down to small sample sizes and resulting low statistical power to find moderately sized differences. However, this lack of significant differences may also reflect the strict inclusion criteria, excluding patients on medication which is anticipated to alter these pathways (and perhaps, as a result, excluding some of the more severely affected HD patients). Our results are at least suggestive that with our patient inclusion criteria, there are differences of any appreciable size in not more than a small proportion of these biological markers, since even in the absence of any such associations a few significant results could readily have been found. We found only very subtle non-significant differences in hypothalamic-pituitary hormones in HD subjects, (e.g. decreased post-sleep GH and fasting DHEAS) that may warrant study in larger controlled cohorts.
